# Supplementary material for: Upconversion Nanoparticles Intercalated in Large Polymer Micelles for Tumor Imaging and Chemo/Photothermal Therapy
Source: Int J Mol Sci. 2023 Jun 24;24(13):10574. doi: 10.3390/ijms241310574 (PMC10342109; doi:10.3390/ijms241310574)
Supplement: Supplementary file 1 [file ijms-24-10574-s001.zip › ijms-2410207-supplementary.pdf]

International Journal of
Molecular Sciences

Supplementary materials

S-1 Spectrum of UCNP photoluminescent

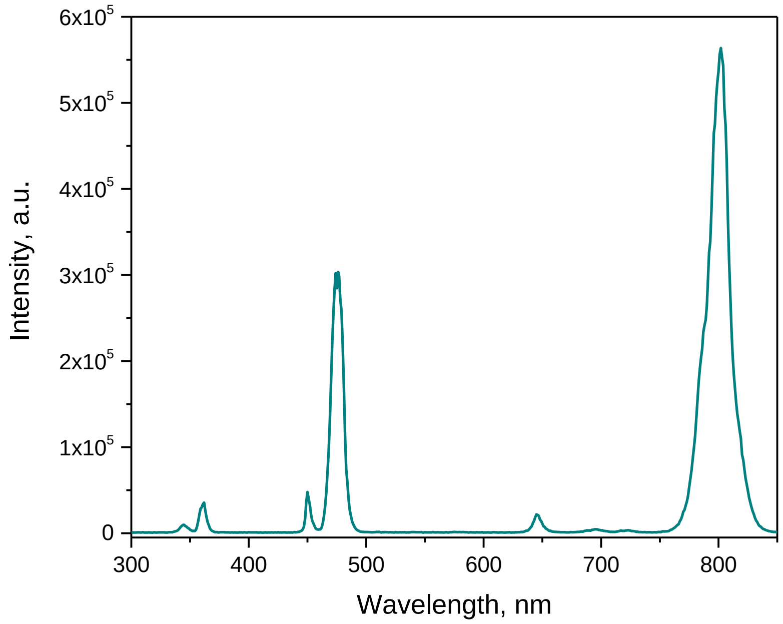

Figure S1. UCNP photoluminescence under 980 nm excitation in hexane.

S-2 FTIR-spectra of UCNP-PMAO and UCNP-PMAO-PVCL

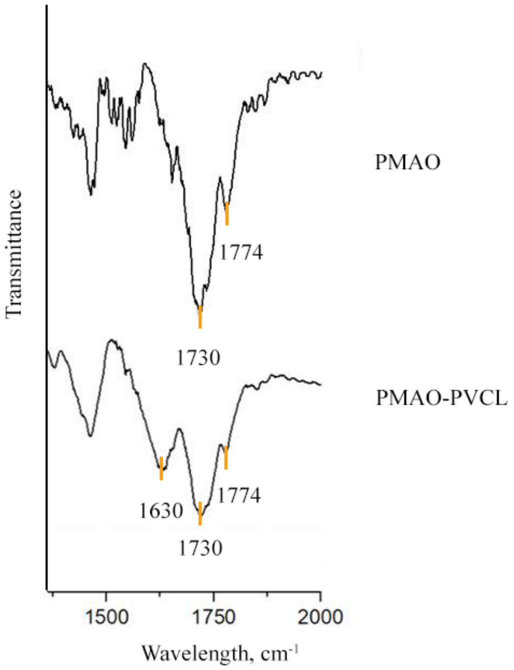

Figure S2. FTIR-spectra of UCNPs modified with PMAO and PMAO-PVCL.

The samples of UCNP-PMAO and UCNP-PMAO-PVCL were dried using a Savant SpeedVac Concentrator (France), thoroughly grounded and then pressed with KBr to form a tablet. FTIR spectra were recorded using a FTIR spectrophotometer (Varian 3100, USA).

A strong stretching vibration of –COO- at 1730 and 1774 cm^{-1} was assigned to carboxyl groups after PMAO hydrolysis. After UCNP-PMAO modification with PVCL, these peaks decreased, indicating complex formation. The presence of PVLC on the surface was confirmed by 1630 cm^{-1} assigned to -C=O group.

S-3 Compound structures

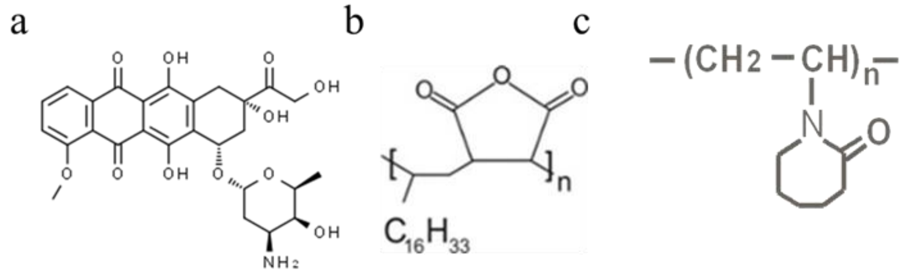

Figure S3. Structures of a) doxorubicin; b) poly(maleic anhydride-*alt*-1-octadecene) (PMAO); c) poly-N-vinylcaprolactam (PVCL)

S-4 Spectrophotometric study of Dox samples in the Ag NP presence

To evaluate the interaction between Ag NPs and Dox, we synthesized Ag NPs by reduction Ag^{+} to Ag^{o} with trisodium citrate using modified method reported by Lee and Meisel [53,54]. Briefly, 50 mL (0.34 mg/mL) silver nitrate ($AgNO_3$) was heated at 80°C for 12–15 min, then 10 mL (0.5 % wt.) trisodium citrate ($Na_3C_6H_5O_7$) was added dropwise and the solution was mixed vigorously for 2 hours at 70°C until color changed to pale yellow. Diameter of prepared Ag NPs was 20±2 nm.

UV–Vis absorption spectra can be used to confirm the assembly of Ag NPs – Dox. Herein, citrate stabilized Ag NPs demonstrate intense absorption peak, also known as LSPR (localized surface plasmons resonance), at λ_{max} ~ 425 nm. In the presence of colloidal Ag NPs, the Dox absorbance raised as the concentration of Ag NPs increased while peak of Dox at 490 nm was obviously blue shifted. Continuous increase and broadening of band at 490 nm suggested that the chemical environment near the Ag NPs has changed (Figure S-4.1). We speculate that a higher concentration of Ag NPs altered the microenvironment around the aromatic residues of Dox. This means that Ag NPs can interact with Dox through amine and hydroxyl groups.

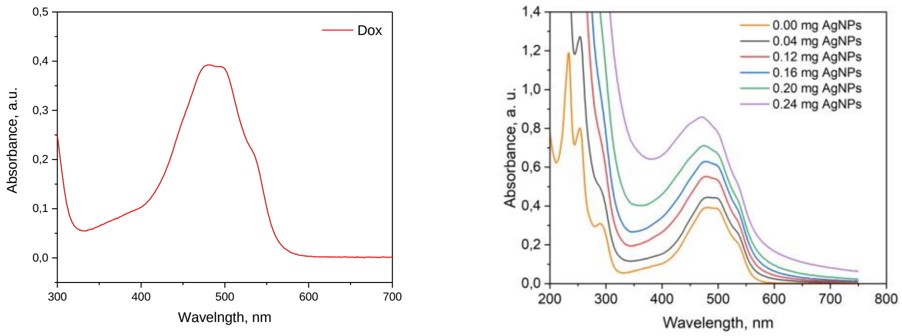

Figure S4.1. UV–Vis absorption spectra of free Dox and Dox with various Ag NP concentrations.

Moreover, emission peaks of Dox appear at 577 and 595 nm under the excitation wavelength of 480 nm, while the absorption band of Ag NPs appears at 426 nm (Figure SI-4.2). The emission spectrum of Dox largely overlaps with absorption spectrum of Ag NPs suggesting the chance of efficient energy transfer between Ag NPs and Dox, where Dox acts as a donor, while Ag NPs act as acceptors.

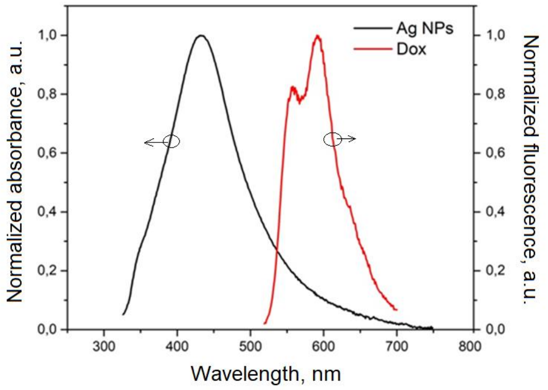

Figure S4.2. The emission spectrum of Dox and absorption spectrum of Ag NPs.

S-5 Free Dox toxicity for MDA-MB-231, Sk-Br-3, and WI-26.

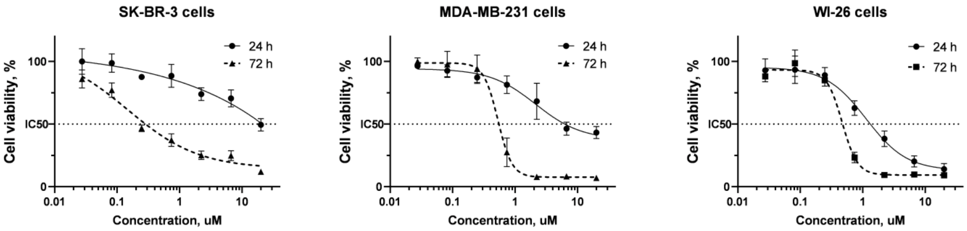

Figure S5. Cell viability of a) human breast adenocarcinoma Sk-Br-3 cells, b) human breast adenocarcinoma MDA-MB-231 cells, and c) human WI-26 fibroblasts under free Dox treatment, 24-h and 72-h incubation, MTT assay. The data are presented as mean ± SD.

S-6 Histology

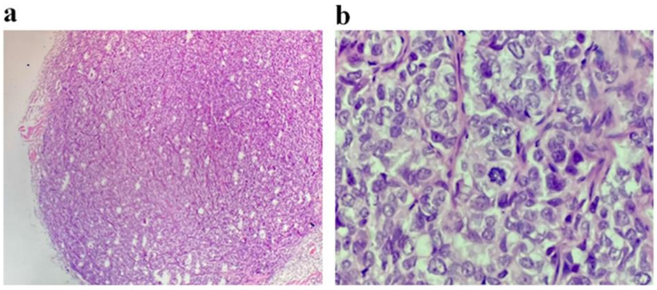

Figure S6. Histological images of the tumor tissue sections stained with hematoxylin and eosin. Control group without treatment a) magnification 40x; b) magnification 400x.

S-7 In vivo studies of UCNP-PMAO-PVCL-Dox injection in comparison with free Dox.

Tumor growth was evaluated throughout the observation period. The *in vivo* treatment efficacy, after a single peritumoral administration to Balb/c nude mice, was assessed by measuring the tumor volumes over a period of 25 days.

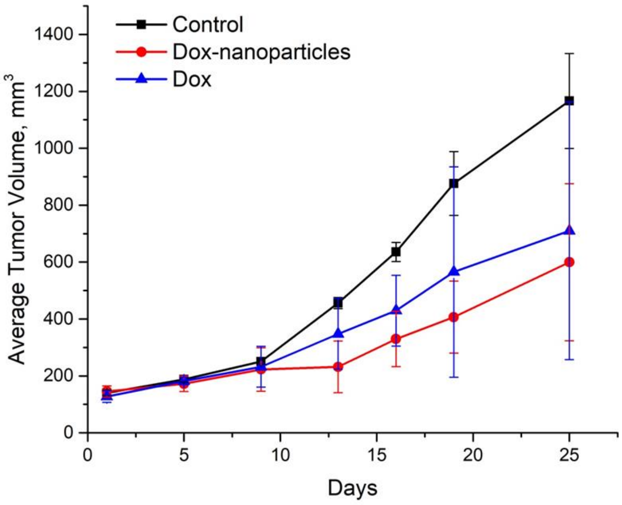

Figure S7. Tumor volume regression in the Balb/c nude mice with Sk-Br-3 human breast cancer xenografts post treatment with UCNP-PMAO-PVCL-Dox and free Dox without NIR-induced treatment.
